# Alteration of the gut microbiota after surgery in preterm infants with necrotizing enterocolitis

**DOI:** 10.3389/fped.2023.993759

**Published:** 2023-01-30

**Authors:** Huijia Lin, Cuifang Xu, Junjin Chen, Xiaolu Ma, Liping Shi, Wei Shi, Lizhong Du, Yan Ni

**Affiliations:** ^1^ Department of NICU, The Children’s Hospital, Zhejiang University School of Medicine, National Clinical Research Center for Child Health, Hangzhou, China; ^2^ Department of National Clinical Research Center, The Children’s Hospital, Zhejiang University School of Medicine, National Clinical Research Center for Child Health, Hangzhou, China

**Keywords:** gut microbiota, necrotizing enterocolitis, preterm, surgery, 16S rRNA sequencing

## Abstract

**Purpose:**

To investigate the dynamic changes in the intestinal microbiota in preterm infants with necrotizing enterocolitis (NEC) before and after treatment *via* a prospective case-control study.

**Methods:**

Preterm infants with NEC and preterm infants with similar age and weight (control group) were enrolled in this study. They were divided into NEC_Onset (diagnosis time), NEC_Refeed (refeed time), NEC_FullEn (full enteral nutrition time), Control_Onset, and Control_FullEn groups according to the time of the fecal material collected. Except for basic clinical information, fecal specimens of the infants were obtained as well at indicated times for 16S rRNA gene sequencing. All infants were followed up after discharge from the NICU, and the growth data of the corrected age of 12 months were acquired from the electronic outpatient system and telephonic interviews.

**Results:**

A total of 13 infants with NEC and 15 control infants were enrolled. A gut microbiota analysis showed that the Shannon and Simpson indices were lower in the NEC_FullEn group than in the Control_FullEn group (*p *< .05). Methylobacterium, Clostridium_butyricum, and Acidobacteria were more abundant in infants with NEC during diagnosis. Methylobacterium and Acidobacteria were remained plentiful in the NEC group until the end of treatment. These bacteria species were significantly positively correlated with CRP and negatively correlated with platelet count. The rate of delayed growth was higher in the NEC group than in the control group (25% vs. 7.1%) at 12 months of corrected age, but there was no significant difference. In addition, the pathways of synthesis and degradation of ketone bodies were more active in the NEC subgroups, including both the NEC_Onset group and the NEC_FullEn group. The pathway of sphingolipid metabolism was more active in the Control_FullEn group.

**Conclusion:**

Even after reaching the full enteral nutrition period, alpha diversity in infants with NEC who underwent surgery was lower than that in the control group infants. It may take more time to reestablish the normal gut flora of NEC infants after surgery. The pathways of the synthesis and degradation of ketone bodies and sphingolipid metabolism might be related to the pathogenesis of NEC and physical development after the occurrence of NEC.

## Introduction

Neonatal necrotizing enterocolitis (NEC) is a life-threatening disease, the typical characteristic of which is intestinal inflammatory necrosis. Most NEC cases are preterm infants (1, 2). The occurring frequency of NEC is close to 7% in very-low-birth-weight infants, and their mortality rates reach one-third. Surgical intervention is done in approximately 20%–40% of NEC infants (3). Also, NEC infants undergoing surgery may experience worse outcomes such as short bowel syndrome and neurodevelopmental dysplasia (4, 5).

The exact pathogenesis of NEC remains undetermined. However, it may involve unusual intestinal bacterial colonization as well as overinflammatory response to the intestinal microbiota (6, 7). Different from the conventional culture-based technique, the gene sequencing of the 16S bacterial ribosomal RNA (16S rRNA) can provide a more detailed spectrum of the human intestinal bacteria (8). Previous studies compared the bacterial abundance between healthy preterm infants and NEC patients. However, most of these studies focused only on the microbiota changes that occurred during the period in which NEC also occurred; in addition, these data did not lead to a unified conclusion (9–12). Some studies focused on the gut microbiota (GM) for surgical NEC. Stewart et al. (13) showed that preterm infants with NEC had a lower diversity and a higher phylum-level Proteobacteria compared with spontaneous isolated perforation using formalin-fixed paraffin-embedded tissue. Correa et al. (14) reported that surgical NEC was associated with a distinct microbiome, which was characterized by low diversity, a higher abundance of Staphylococcus, and Clostridium_*sensu*_*stricto* when compared with infants who received intestinal surgery without NEC. Different from these two researchers, Brower-Sinning et al. (15) found that no significant difference was observed in the mean Shannon diversity index (SDI) of NEC and non-NEC groups. They also concluded that NEC and non-NEC subjects had high interindividual variability and an abundance of opportunistic pathogens.

Different from previous studies, we designed this prospective case-control study to examine intestinal microbiota alteration in NEC between the time of diagnosis and the end of treatment. Thus, the purpose of this study is not only to investigate the abundance, composition, and distribution of the intestinal microbiota in NEC infants, but also to observe the alterations in the intestinal bacterial spectrum during treatment to provide scientific data for clinical pediatricians for NEC treatment.

## Methods

### Study design and patients

It was a prospective monocentric case-control cohort study conducted from June 2018 to June 2020. This project was authorized by the Ethics Committee of the Children's Hospital of Zhejiang University School of Medicine (No.2021-IRB-210).

The inclusion criteria of the NEC group were as follows:

1. The gestational age (GA) of infants was required to be ≤34 weeks and the birth weight (BW) of infants should be ≤2000 g.
2. Infants who were diagnosed with NEC at Bell's stage II or III based on the modified Bell's staging (16).
3. Infants who received surgical intervention in the form of either enterostomy or anastomosis.

NICU physicians invited neonatal surgeons for consultations and then made a collective decision on surgical intervention. The surgical indication of NEC was the presence of pneumoperitoneum (Bell stage IIIa) and persistent ileus or abdominal distension (persistent Bell stage IIb > 24 h), coupled with deteriorating clinical and biochemical statuses (e.g., either shock or a decrease in platelet/neutrophil count, or both, or persistent metabolic acidosis) (3).

The exclusion criteria of the NEC group were as follows:

1. Infants who had serious malformations, including congenital intestinal malformations.
2. Infants who did not have complete clinical data and/or failed to collect or store samples by following the necessary procedures.

The inclusion criteria of the control group were as follows:

Infants whose GA and BW were ≤34 weeks and ≤2000 g, without either NEC or congenital intestinal malformations, were included as controls.

### Groups and fecal sample gathering

Fecal samples were gathered at three time points for the NEC group: NEC diagnosis or surgery at acute phase, 24 h within refeed after surgery, and the day of achieving full enteral nutrition. Thus, NEC groups were divided into three subgroups based on the fecal sample collection, the NEC_Onset group, the NEC_Refeed group, and the NEC_FullEn group, respectively.

Because NEC usually occurred 2 weeks after birth, the first time that the sample was collected for the control group was during this period. The second-time sample collection for this group was on the day of achieving full enteral nutrition. Thus, the controls were divided into the Control_Onset and the Control_FullEn groups as well.

The fecal samples were obtained by using disposable sterilized spatulas from the diaper during diaper exchange or from the ostomy bag of the infants who received ostomy. The collected samples were then immediately stored in a −80°C freezer until they were sent to Novogene Co. Ltd. (Headquarters), Beijing, China, for 16S rRNA analysis.

### Data collection and definition

The data pertaining to hospitalization (including demographics, management or treatment, and laboratory examination) were collected from the patient charts or electronic systems, and all infants were followed up in the preterm infant outpatient department and by telephonic interviews after discharge. Growth parameters such as weight, height, and head circumference (HC) were obtained from clinical medical records or from their parents.

Growth delay was defined as weight, height, or HC less than 10 percentile at 12 months of corrected age, using WHO Child Growth Standards (2006) (17). Those infants who underwent a surgical treatment and achieved full enteral nutrition after treatment were said to have received successful clinical cure.

### Microbiota analysis

Genomic DNA was isolated by using CTAB/SDS technology, purified on a 1% agarose gel, and adjusted to a concentration of 1 ng/μl by using sterile water. The 16S rRNAs were amplified with V4 region primers (515F and 806R) with barcode. PCR was reacted in a 30 μl mixture consisting of a 15 μl Phusion *R* High-Fidelity PCR Master Mix (New England Biolabs, Ipswich, MA, USA), 0.2 μM of 515F and 806R primers, and approximately 10 ng of reversed DNA. Thermal cycling was done for 1 min at 98°C, repeated 30 cycles at 98°C for 10 s, at 50°C for 30 s, and at 72°C for 30 s, and finally heated at 72°C for 5 min to terminate the reaction. The PCR-reacted mixture was then diluted with a 30 μl loading buffer that contained SYB green and isolated on a 2% agarose gel by electrophoresis. The PCR band (PCR products) was cut off and mixed with an equal volume of gel extraction buffer and purified with a PCR purification kit, GeneJETTM Gel Extraction Kit (Thermo Scientific, Waltham, MA, USA). Next, Ion Plus Fragment Library Kit 48 rxns (Thermo Scientific) was employed to prepare the sequencing libraries, the quality of which was analyzed on a Qubit@2.0 Fluorometer (Thermo Scientific). Lastly, the libraries were sequenced on the Ion S5TM XL System to generate single-end reads with 400–600 bp.

For amplicon sequences, raw data were processed using Qiime2 pipeline (2020 version) (18). FastQC was initially applied for quality evaluation. Dada2 (19) was used to denoise sequences and generate ASVs (amplicon sequence variants). ASVs were then mapped by using the Silva 132 database with 99% identity for taxonomy annotation. PICRUSt2 (2.2.0_b) (20) was further applied for predicting metagenome functions.

### Statistical analysis

All clinical data analyses were performed with SPSS software (IBM-SPSS, Chicago, IL, USA; version 16.0). Based on the distribution pattern, skewed or otherwise, continuous variable data were summarized as the average ± standard deviation (SD). Comparisons of continuous variables were done with Student’s *t*-test for the NEC and control groups. The repeated measures ANOVA was applied for making comparisons within the NEC subgroups. Pearson's chi-square test was employed for comparing categorical variables. A value of *p *< .05 was considered statistically significant.

A statistical analysis of the intestinal microbiota was performed using R software 3.6.3. The Shapiro–Wilk test was used to test for normality. Variables that were normally distributed were examined using ANOVA and Student’s *t*-test, while those that were not normally distributed were assessed using the Kruskal–Wallis and Mann–Whitney *U* tests.

The differences in the intestinal microbiota between the two groups were examined by using either the Student’s *t*-test or the Mann–Whitney *U* test. ANOVA or the Kruskal–Wallis test was applied for making three group comparisons. The alpha diversity indices (the Shannon Diversity index and Simpson index) were calculated to measure the diversity of the GM using Vegan packages. Spearman’s rank correlation coefficient analysis was employed to evaluate the correlations between the GM and the clinical markers, and it was visualized using a heatmap.

## Results

### Study design and clinical characteristics

**Figure 1:**
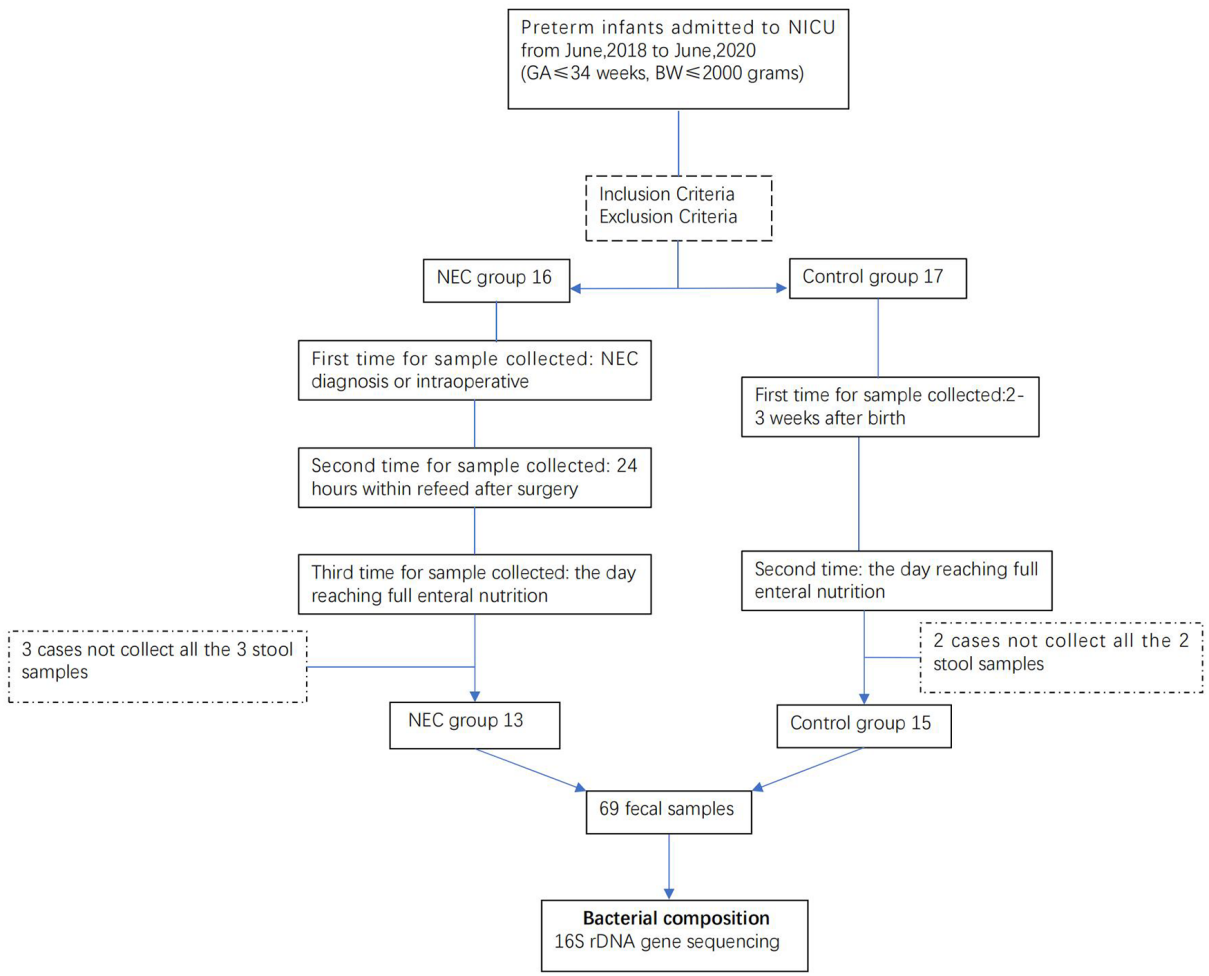
Flow diagram.

In this study, 13 infants with NEC (NEC group) and 15 similar GA and BW infants (control group) were enrolled for analysis. All 13 infants received surgical intervention, of which 11 underwent enterostomy and the remaining 2 anastomosis. A total of 39 and 30 fecal samples were collected from the NEC and the control groups, respectively, for microbiota analysis. The whole process is represented as a flow diagram (Figure 1).

The mean GA of the NEC group was 30.5 ± 2.4 weeks and that of the control group was 30.6 ± 2.2 weeks, separately. Mean BWs were 1428 ± 327 g and 1256 ± 208 g, respectively, in these two groups. The age of admission in the NEC group was much higher than in the control group. Also, antibiotic use was significantly higher in the NEC group (100.0% vs. 26.7%, *p *< .001). However, no significant differences in terms of the mean BW, mean GA, gender ratio, delivery mode, or type of feed were observed in these two groups (Table 1).

**Table 1:** Demographic and clinical characteristics.

Items	NEC group	Control group	*p*
Cases (*n*)	13	15	–
GA (weeks) (mean ± SD)	30.5 ± 2.4	30.6 ± 2.2	.910
BW (g) (mean ± SD)	1428 ± 327	1256 ± 208	.120
Male (*n*, %)	5 (38.5)	4 (26.7)	.505
SGA (*n*, %)	3 (23.1)	4 (26.7)	.827
Age at admission (days), median (IQR)	15 (7,20)	2 (1,5)	.001*
**Delivery mode**			
C section (*n*, %)	10 (76.9)	10 (66.7)	.549
Twins (*n*, %)	4 (30.7)	8 (53.3)	.229
Antibiotic use (*n*, %)	13 (100.0)	4 (26.7)	<.001*
Age at onset of sample collection (days), median (IQR)	17 (13,29.5)	15 (13,17)	.220
Age at refeed of sample collection (days), median (IQR)	26 (23,37.5)	–	–
Age at full EN of sample collection (days), median (IQR)	54 (43,63.5)	33 (29,42)	.002*
**Type of feeding**			
Breast milk (*n*, %)	7 (53.8)	11 (73.4)	.283
Formula (*n*, %)	4 (30.8)	2 (13.3)	.509
Mixed feeding (*n*, %)	2 (15.4)	2 (13.3)	.877
Age of full EN (days) (mean ± SD)	59 ± 18	37 ± 9	.001*
Growth delay (*n*, %)§	3 (25.0)	1 (7.1)	.288

Note: NEC, necrotizing enterocolitis; GA, gestational age; BW, birth weight; SD, standard deviation; SGA, small for gestational age; IQR, interquartile range; EN, enteral nutrition; § NEC group *n* = 11, control group *n* = 14.

^*^ *p* < .05.

With regard to the time of fecal collection, there were four infants in the NEC group whose feces were gathered during surgery the first time. The mean interval between NEC diagnosis and surgery was 2(1,4) days. The age of onset for fecal collection was similar in both groups. However, as the time for achieving full enteral nutrition in the NEC group was significantly longer than that in the control group (*p *= .001), the time for fecal collection in this group was also longer than that in the other group (*p *= .002).

### Surgical findings for the NEC group

Surgical findings are described in Table 2. A perforated bowel was found in three patients and necrosis without perforation was found in the remaining ten. The most frequent site of NEC was located in the ileum. Two patients received enterostomy and the others received primary anastomosis. All underwent a pathological examination (Supplementary Figure S1).

**Table 2:** Surgical findings for infants with NEC.

Case	Age at NEC diagnosis (days)	Age of surgery (days)	Type of injury	Location	Extent of disease	Surgical intervention
1	15	17	Perforated bowel	Ileum	Multifocal	Enterostomy
2	26	27	Necrosis without perforation	Ileum + colon	Multifocal	Primary anastomosis
3	30	42	Necrosis without perforation	Ileum + colon	Multifocal	Enterostomy
4	9	10	Necrosis without perforation	Ileum + colon	Multifocal	Primary anastomosis
5	16	17	Necrosis without perforation	Ileum	Multifocal	Primary anastomosis
6	11	28	Necrosis without perforation	Jejunum + ileum + colon	Multifocal	Primary anastomosis
7	16	17	Necrosis without perforation	Ileum + colon	Multifocal	Primary anastomosis
8	15	17	Necrosis without perforation, Pneumatosis	Ileum + colon	Multifocal	Primary anastomosis
9	15	32	Necrosis without perforation	Ileum + colon	Multifocal	Primary anastomosis
10	8	9	Necrosis without perforation	Ileum	Multifocal	Primary anastomosis
11	39	41	Perforated bowel	Ileum + colon	Multifocal	Primary anastomosis
12	14	20	Perforated bowel	Ileum + colon	Multifocal	Primary anastomosis
13	27	28	Necrosis without perforation	Ileum	Multifocal	Primary anastomosis

NEC, necrotizing enterocolitis.

Taking into account the interval between NEC diagnosis and surgery, all infants with NEC were divided into two subgroups, NEC_SI(NEC_short interval) group including patients 3, 6, 9, and 12 and NEC_LI(NEC_long interval) group including the remaining 9.

### Clinical data between the NEC and the control groups

Two infants were lost to follow-up after discharge. One belonged to the NEC group and the other to the control group. In addition, one infant with NEC died after discharge before 12 months of corrected age and one developed short bowel syndrome. Therefore, after 12 months of physical development, there were 11 NEC infants and 14 control infants, respectively. The rate of growth delay at the corrected 12 months was higher in the NEC group than in the control (25.0% vs. 7.1%); however, no statistically significant difference was found between the groups (Table 1).

### Laboratory tests among the groups

The laboratory tests were also compared among the different subgroups. C-reactive protein (CRP) was obviously higher in the NEC_Onset group than in the Control_Onset group, and the platelet count was significantly lower in the NEC_Onset group (*p *< .001, *p *< .001). After reaching the full enteral nutrition period, CRP and the platelet count showed no remarkable differences between the NEC_FullEn and the Control_FullEn subgroups (*p *> .05). However, CRP and platelet showed significant differences among the three subgroups of NEC.

In addition, the total protein and albumin levels were slightly lower in the NEC_Onset group than in the other groups. Much increased aspartate aminotransferase (AST), alanine aminotransferase (ALT), and direct bilirubin (DB) levels were found in the NEC_FullEn group than in the Control_FullEn group (*p *< .05) (Table 3).

**Table 3:** Laboratory examinations among different subgroups.

Laboratory examination	NEC_Onset (*n* = 13)	NEC_Refeed (*n* = 13)	NEC_FullEn (*n* = 13)	Control_Onset (*n* = 15)	Control_FullEn (*n* = 15)	*p*_1_	*p*_2_	*p*_3_
WBC (*10^9^/L)	9.3 ± 4.9	13.2 ± 3.3	11.1 ± 3.7	12.3 ± 3.5	10.9 ± 3.0	.073	.853	.197
Hbg (g/L)	134 ± 22	126 ± 23	137 ± 28	154 ± 17	136 ± 27	.013*	.906	.675
L (*10^9^/L)	2.4 ± 1.2	4.8 ± 2.4	5.4 ± 2.0	5.0 ± 1.6	5.7 ± 1.5	<.001*	.589	.007*
N (*10^9^/L)	5.4 ± 3.9	6.2 ± 2.1	4.0 ± 2.0	5.1 ± 2.0	3.5 ± 2.1	.818	.497	.100
PLT (*10^9^/L)	118 ± 74	248 ± 184	288 ± 129	278 ± 122	351 ± 136	<.001*	.226	.002*
CRP (mg/L)	84.24 ± 38.06	110.96 ± 9.05	1.76 ± 1.67	0.64 ± 0.44	0.63 ± 0.61	<.001*	.055	<.001*
TP (g/L)	39.0 ± 4.7	37.4 ± 4.6	41.1 ± 2.4	43.6 ± 3.9	43.6 ± 3.7	.010*	.048*	.130
Albumin (g/L)	25.9 ± 3.9	25.6 ± 5.1	31.2 ± 2.8	30.4 ± 3.2	31.7 ± 2.5	.003*	.632	.002*
Globulin (g/L)	12.9 ± 3.8	11.8 ± 1.9	9.9 ± 1.9	13.2 ± 2.2	11.9 ± 2.5	.828	.028*	.010*
ALT (U/L)	11 ± 4	60 ± 55	72 ± 52	7 ± 4	9 ±± 2	.069	.017*	.053
AST (U/L)	40 ± 17	147 ± 137	136 ± 124	24 ± 16	24 ± 7	.070	.011*	.119
DB (μmol/L)	27.6 ± 18.2	42.7 ± 31.3	64.5 ± 57.8	11.9 ± 2.5	13.0 ± 10.0	.068	.008*	.010*
IB(μmol/L)	71.5 ± 40.6	58.9 ± 51.3	76.4 ± 63.7	99.6 ± 30.3	60.0 ± 43.2	.046*	.362	.763
Scr (μmol/L)	61 ± 20	44 ± 11	42 ± 10	67 ± 13	51 ± 1034	.392	.022*	.008*
Urea (mmol/L)	5.34 ± 1.98	3.43 ± 1.67	3.37 ± 1.35	4.47 ± 2.35	3.58 ± 1.28	.301	.678	.005*

Note: Hbg, hemoglobin; L, lymphocyte; N, neutrophil; CRP, C-reactive protein; PLT, platelet count; TP, total protein; ALT, aspartate aminotransferase; AST, alanine aminotransferase; DB, direct bilirubin; IB, indirect bilirubin; Scr, serum creatinine; Urea, urea nitrogen; *p*_1_, comparison between NEC_onset and control_onset groups; *p*_2_, comparison between NEC_FullEn and control_FullEn groups; *p*_3_, comparison among NEC_onset, NEC_refeed, and NEC_FullEn groups; NEC, necrotizing enterocolitis.

* *p* < .05.

### The GM feature of the NEC group

The species cumulative curve showed an asymptote after a sharp rise and tended to be flat, indicating that the sequencing results were adequate to reflect the diversity in the sample (Supplementary Figure S2). Furthermore, the samples were examined by using principal coordinate analysis (PCoA), which is based on the Jaccard index, to determine the microbiota structure. The NEC_Onset group and the control_Onset group showed different types of clustering (Figure 3E), but the distribution of each sample was relatively scattered. When all infants achieved full enteral nutrition, the two groups also showed separation, while the control group was more dispersed (Figure 4E).

**Figure 2:**
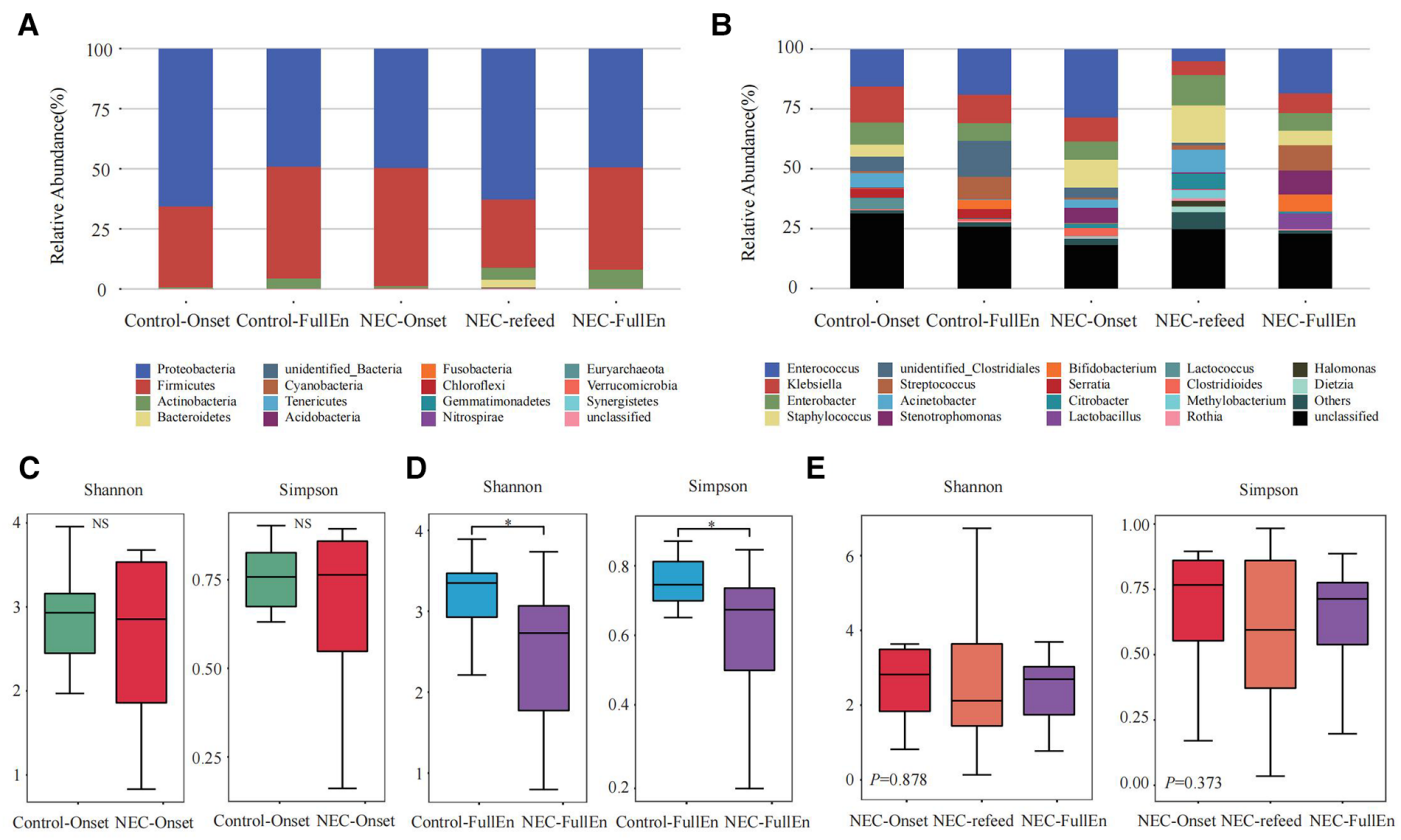
Taxonomic composition analysis and alpha diversity analysis. (**A,B**) Taxonomic composition analysis. (**A**) The top 10 at the phylum level between each group. (**B**) The top 20 at the genus level between each group. (**C–E**) Alpha diversity analysis. (**C**) Alpha diversity analysis between the NEC_Onset group and the Control_Onset group. (**D**) Alpha diversity analysis between the NEC_FullEn group and the Control_FullEn group. (**E**) Alpha diversity analysis between the three subgroups.

**Figure 3:**
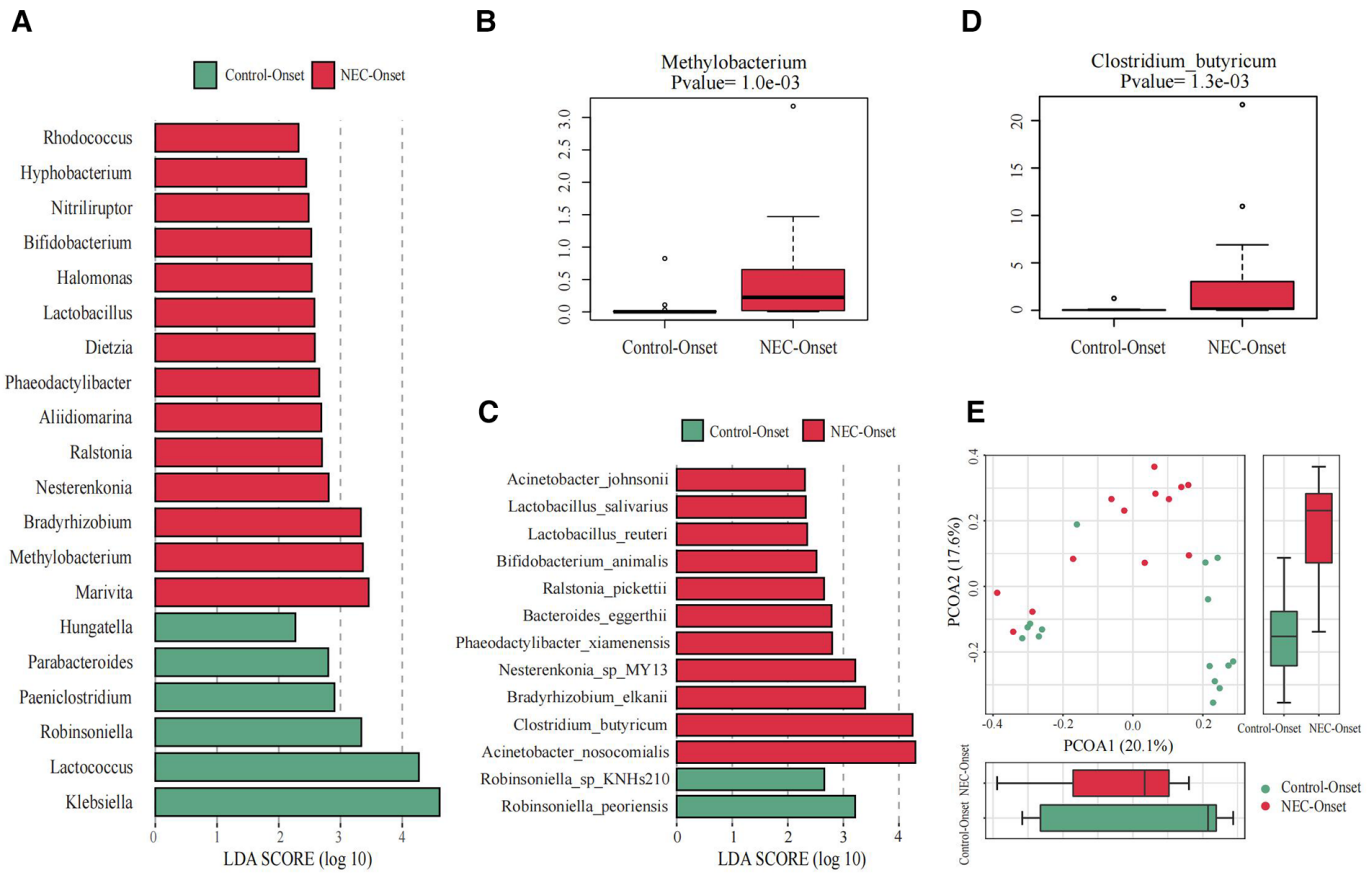
Significant differences in the microbiota and correlation. (**A**) Gut microbiota analysis at the genus level using the LDA score for NEC group infants and control group infants. (**B**) Bar chart of Methylobacterium between the NEC_Onset group and the Control_Onset group. (**C**) Gut microbiota analysis at the species level using the LDA score for NEC group infants and control infants. (**D**) Bar chart of Clostridium_butyricum between the NEC_Onset group and the Control_Onset group. (**E**) Principal coordinate analysis (PCoA) of the microbial communities of the two groups. The microbiota cluster in the infants of the NEC_Onset group (red circle) and the Control_Onset group (green circle) was examined by using PCoA. NEC, necrotizing enterocolitis; LDA, linear discriminant analysis.

**Figure 4:**
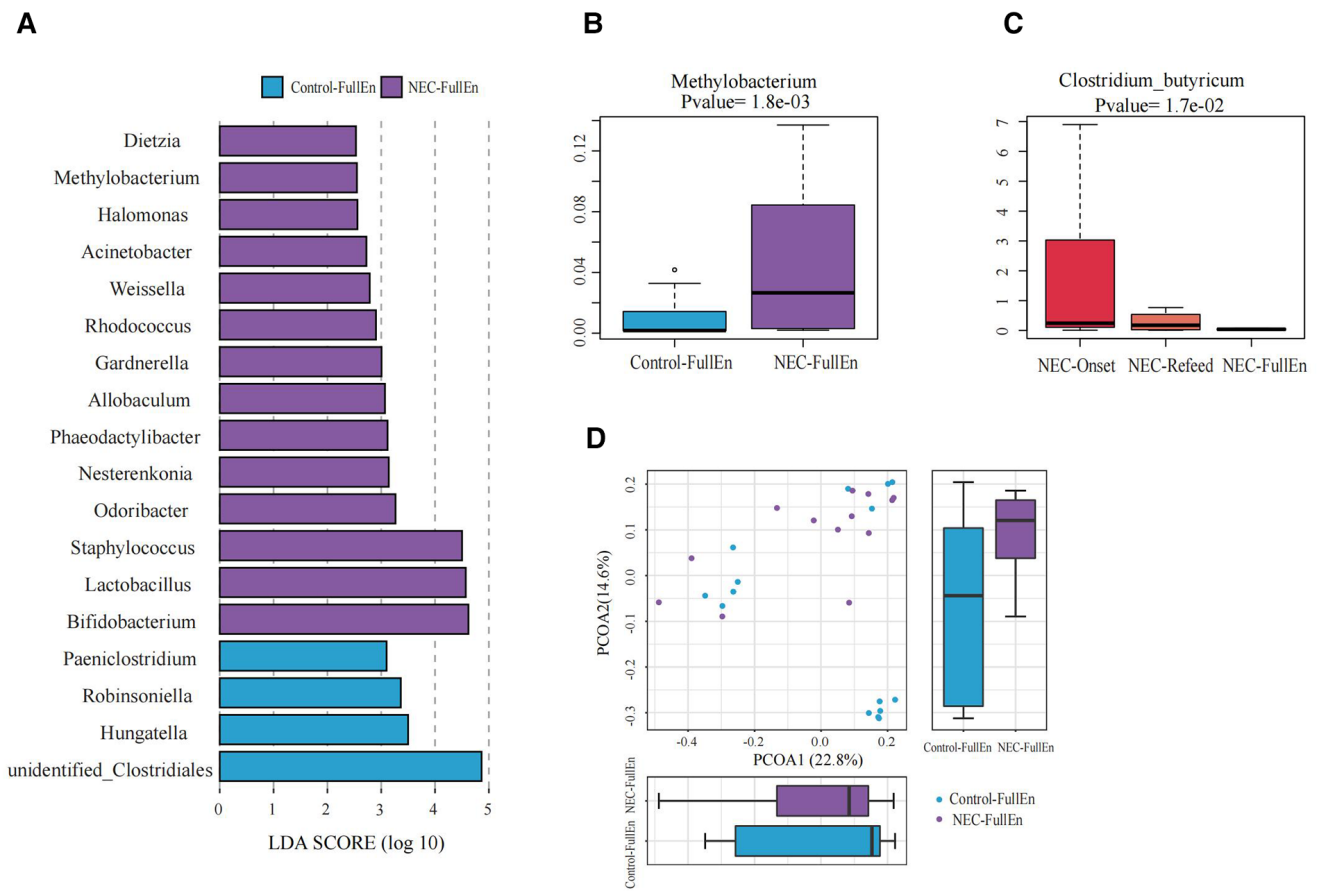
Significant differences in the microbiota and correlation. (**A**) Gut microbiota analysis at the genus level using the LDA score between two groups after they achieved full enteral nutrition. (**B**) Bar chart of Methylobacterium between the NEC_FullEn group and the Control_FullEN group. (**C**) Bar chart of Clostridium_butyricum among the three subgroups of NEC. (**D**) Principal coordinate analysis (PCoA) of the microbial communities of the two groups after achieving full enteral nutrition. The microbiota cluster in the infants of the NEC_FullEn group (purple circle) and the Control_FullEn group (blue circle) was examined by using PCoA. NEC, necrotizing enterocolitis; LDA, linear discriminant analysis.

In the examination of the overall GM spectrum, the Firmicutes and Proteobacteria were found to be the most abundant flora at the phylum level among all subgroups, which constituted more than 92% of the total bacteria species among the groups. In addition, at the genus level, *Enterococcus*, *Klebsiella*, *Enterobacter*, and *Staphylococcus* were the most abundant flora in each group (Figures 2A,B).

An analysis of the alpha diversity index indicated that the Shannon and Simpson indices of the NEC_Onset group were slightly lower than those of the Control_Onset group; however, there was no obvious difference between the groups (Figure 2C). These two indices in the NEC_FullEn group were much lower than those in the Control_FullEn group when the infants achieved full enteral nutrition (*p *< .05) (Figure 2D). The alpha diversity index was also compared between the three subgroups of NEC infants. The Shannon and Simpson indices first showed a declining trend and then a rising trend after NEC treatment. Nevertheless, statistical analysis showed that the alpha diversity index was not significantly different over time (*p *= .878, *p *= .373) (Figure 2E).

### Differential GM and correlations with clinical markers

The differences at each taxonomic level were observed through LEfSe analysis as well. Firmicutes and Proteobacteria were the most abundant microbiota at the phylum level in all subgroups. There were no differences in the amount of Firmicutes and Proteobacteria between the NEC_Onset and the Control_Onset groups, as well as the NEC_FullEn and the Control_FullEn groups. However, the relative amounts of Acidobacteria at the phylum level were remarkably higher in the NEC_Onset group than in the Control_Onset group (*p *= .0015). Notably, after treatment, there was an abundance of Acidobacteria in the NEC_FullEn group (*p *= .00036). Although there was a gradual declining trend of Acidobacteria among the NEC subgroups during therapy, no statistical significance was found (*p *= .178). In addition, Methylobacterium at the genus level and Clostridium_butyricum at the species level were also significantly abundant in the NEC_Onset group (Figures 3A–D). Even after treatment, the amount of Methylobacterium was still higher in the NEC_FullEn group (Figures 4A,B). In addition, there was a gradual decrease in the amount of Clostridium_butyricum among the three subgroups of infants with NEC (Figure 4C).

**Figure 5:**
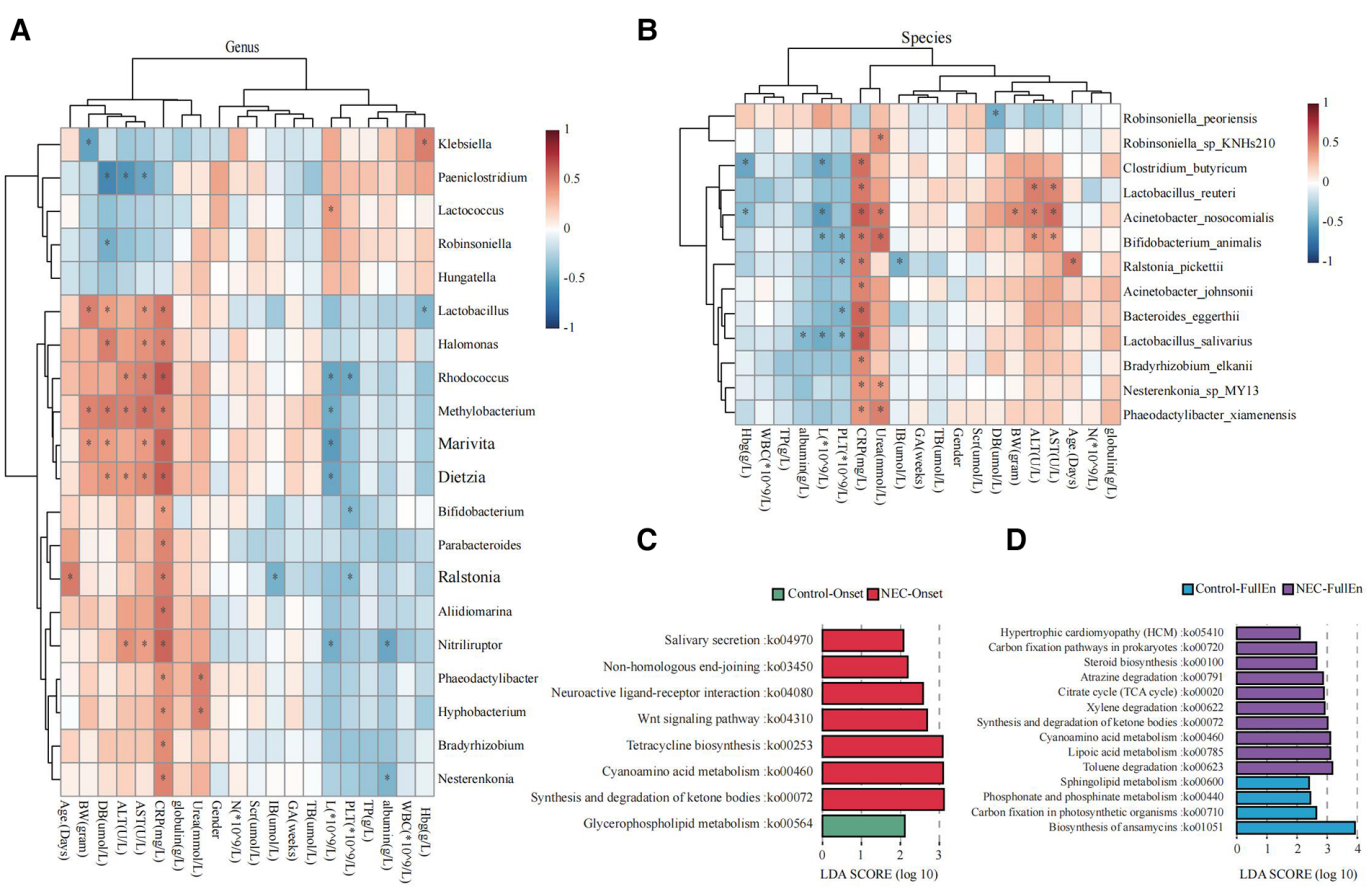
Microbial function prediction. (**A**) Correlation heatmap for bacteria at the genus level correlated with clinical characteristics and examinations. (**B**) Correlation heatmap for bacteria at the species level correlated with clinical characteristics and examinations. (**C**) LDA score for microbial pathways when NEC occurred. (**D**) LDA score for microbial pathways when all infants achieved full enteral nutrition. NEC, necrotizing enterocolitis; LDA, linear discriminant analysis.

Inflammatory reaction always co-occurs with NEC. After a laboratory examination, there is a change in inflammatory indicators during NEC diagnosis or treatment. So, in this study, we also examined the fecal species at different levels correlated with infants’ characteristics and tests. Our examination revealed that Methylobacterium and Clostridium_butyricum were significantly positively correlated with CRP and negatively correlated with platelet count (Figures 5A,B).

### Metabolic functions prediction by differential GM

Increased activities of the pathways related to ketone body synthesis and degradation, metabolism of cyanoamino acid, tetracycline biosynthesis, and Wnt signaling were found in the microbiota of the NEC_Onset group rather than that of the Control_Onset group (Figure 5C). The active difference in the ketone body synthesis and degradation pathway was the most significant factor between the two groups. Notably, this pathway was still more active in NEC infants than those in the control infants after treatment (Figure 5D). Interestingly, the pathway of sphingolipid metabolism was more active in the Control_FullEn group than in the NEC_FullEn group after treatment.

## Discussion

NEC is a devastating disease that occurs predominantly in premature infants who survive in the preliminary days after birth (21). Although the precise etiology and pathogenesis of NEC is unclear to date, abnormal microbial colonization might be one of the important risk factors related to this disease (22). Some studies on the alteration of the intestinal microbiota prior to NEC symptoms after birth (10, 11) have shown that the intestinal diversity undergoes a change before clinical symptoms occur (23). Some specific intestinal bacteria have been described in different reports; however, there is no universal conclusion on NEC infants, and such conclusion may disclose only some patient variation but not the real disease etiology (24–26). Unlike these research studies, our longitudinal study collected a serial fecal sample from the time of NEC diagnosis to investigate the changes in the intestinal microbiota after NEC treatment. In addition, our study attempted to observe whether the GM had been restored when infants achieved full enteral nutrition as NEC was clinically cured.

Some previous studies (24, 27, 28) showed that the intestinal diversity decreased before NEC occurred. Some studies (29–31) did not even show any significant difference between the NEC and the control groups. Our results indicated that the Shannon and Simpson indices did not show any significant differences when NEC occurred; however, these two indices were lower in NEC infants after treatment. As the Shannon and Simpson indices are believed to reflect the abundance and evenness of the microbiota, the lower diversity may be associated with a decreased variety of bacteria species. Our results have demonstrated that the alteration of the GM diversity is closely correlated with the initiation and progression of NEC. Even at the time of achieving full enteral nutrition and when NEC infants were clinically cured, the GM alpha diversity had not yet recovered when compared with the control group. For preterm infants, more time is required for the establishment of intestinal flora because of many risk factors (32). Because infants with NEC underwent surgery, more time might be needed for the reestablishment and restoration of gut flora.

Given that the major gut bacteria in the preterm infants were Proteobacteria, Bacteroidetes, Firmicutes, and Actinobacteria at the phylum level were found to be similar to those in the previous report (26, 33). Through LEfSe analysis, our results indicated that Acidobacteria at the phylum level were significantly different between the NEC and the control groups. Even though there was a gradual decrease in this species after NEC therapy, it was still higher than that in control infants. Acidobacteria are the most distributed bacterial phyla, and they are the richest bacteria among a series of ecosystems, especially in soils. However, there are only limited studies on Acidobacteria in intestinal disease. Igarashi et al. (34) reported that the presence of the microbiota in gastric fluid could be described as Bacteroidetes > Proteobacteria, but Acidobacteria were non-existent in functional dyspepsia patients. This indicated an alteration of Acidobacteria in gastrointestinal disorders. Genome sequencing and metabolic analysis revealed some functionalities of Acidobacteria in preterm infants, such as carbohydrate metabolism, nitrogen metabolism, formation of exopolysaccharides, and transport (35). But it was unclear how Acidobacteria influenced the development of NEC.

In addition, our data revealed that the amount of Methylobacterium at the genus level was higher in NEC infants. Methylobacterium belongs to the family of Proteobacteria. Previous studies also reported similar results. Wang et al. indicated that an increased amount of *γ*-proteobacteria accompanied by a reduction of other bacterial species was found in patients with NEC (28). In a prospective case-control observation, Barbara et al. showed that before the occurrence of NEC, there was a relative enrichment of *γ*-proteobacteria in infants with very low BW (33). The findings of Lindberg et al. also indicated the association between NEC and gut Proteobacteria (36).

Clostridium_butyricum belongs to the family of anaerobic spore-forming bacillus, a gram-positive bacterium. It can produce a large amount of butyric acid. In recent years, some research studies indicated that the abnormality of Clostridium_butyricum was related to NEC onset (25, 37). Hosny et al. (38) reported that Clostridium_butyricum was significantly higher in excrement specimens collected from infants with NEC than in those taken from controls in the Neonatal Intensive Care Unit (NICU) from southern France. Dong et al. (39) also reported that 15 of 37 (40.50%) samples were contaminated with Clostridium_butyricum during an NEC outbreak at a hospital in China. They reasoned that the NEC outbreak was epidemiologically associated with the contamination of this bacterial species in the hospital. Our results on Clostridium_butyricum were consistent with those of previous studies and indicated that this species was related to NEC development.

Through Spearman correlation analysis, we observed a clear correlation between Methylobacterium, Clostridium_butyricum, Acidobacteria, increased CRP, and decreased platelets. CRP and platelet counts were the most commonly measured biomarkers of inflammation and were related to the severity of NEC. Severe thrombocytopenia (<100 × 10^9^/L) was considered as a predictor of bowel gangrene, morbidity, and mortality (40, 41). Similar to thrombocytopenia, persistently elevated CRP in NEC infants made a case for surgical intervention (42). Therefore, in this respect, the abnormalities of these bacteria suggest a strong correlation with NEC. In addition, our data suggested that an excessive quantity of a single bacterium was not the cause of NEC, and that NEC might be caused by the combinative effect of multiple bacteria. In fact, a further study is necessary to know about this combined effect.

Through functional prediction analysis, we identified that the pathway of ketone body synthesis and degradation was more significantly active in infants with NEC, irrespective of whether NEC occurred before or after therapy. Ketone bodies consist of three different small molecules, acetoacetate, acetone, and *β*-hydroxybutyrate (*β*-HB), which are converted into main brain energy substrates during prolonged fasting or starvation. Recent studies indicate that ketone body signaling and ketone bodies themselves are associated with intestinal homeostasis and intestinal microbiota. *β*-HB, one of the ketone bodies, acts by inhibiting class I histone deacetylases to activate Notch signaling, thereby guiding the self-renewal and pedigree of intestinal stem cells (43). Other analyses suggest that ketone bodies selectively inhibit bifidobacterial growth, resulting in decreased intestinal Th17 cell levels (44). The Th17 cell plays a crucial role in autoimmune and abnormal inflammatory responses in the mucosal surfaces of the gastrointestinal tract (45). They are pro-inflammatory because they secrete cytokines that promote inflammation. On the other hand, under a stable state, the Th17 cells secrete both IL-22 and IL-17 and occupy mucosal barriers to regulate local homeostasis by promoting the function of the epithelial protective screen (46). Based on our data, we consider that the pathway of synthesis and degradation of ketone bodies might be associated with NEC onset and development. An in-depth exploration of how the intestinal microbiota participates in this pathway, such as combined intestinal metabolism (26), may help us understand the pathogenesis of NEC and devise ways to provide better treatment.

After discharge, NEC infants need to be followed up for physical development and neurodevelopmental outcomes (47). Our previous study (48) presented that physical development would be adversely affected in NEC infants, particularly in those who received surgical treatments. Similar results were also demonstrated in other research studies (49, 50). Our data also showed that growth delay occurred more frequently in the NEC group, although there was no significant difference with the control groups. Functional prediction analysis revealed that the pathway related to sphingolipid metabolism was more active in the control group when the infants achieved full enteral nutrition. Because of the multiple variations in the structural component, sphingolipids are extremely multiplexed lipids. They not only construct cellular membranes, as crucial signaling factors sphingolipids also participate in the diversity of cellular function regulations, such as cellular proliferation, growth and differentiation, as well as apoptotosis (51). Recent research studies indicated that intestinal sphingolipids played a crucial role in immunity and inflammatory disorders (52). They were also beneficial for maintaining gut homeostasis and symbiosis (53). Thus, combined with the results of our study and the research concerning the function of sphingolipids, we consider that the pathway of sphingolipid metabolism may act as functions related to physical development. Clearly, more further studies are needed to focus on this.

Among NEC patients, four infants had a relatively long interval between diagnosis and operation. It is valuable to explore and compare the GM features of NEC patients who had long or short intervals from diagnosis to surgery (NEC_LI vs. NEC_SI). The Shannon and Simpson indices indicated that microbial diversity was comparable between the two groups at three different time points (Supplementary Figure S3A). A further LEfSe analysis showed the differential bacteria at the genus level between two subgroups, especially at the refeed time point. Six different genera, including *Sphingomonas*, *Gardnerella*, and *Bacteroides*, were more abundant in the NEC_SI group (Supplementary Figure S3B). However, the small sample size of the two subgroups in this study requires further validation.

## Limitation

The limitations of this study are its small sample size, single-center design, non-performance of fecal metabolome examination, and a non-analysis of fecal samples after discharge. Although in our study, the control group was age- and gender-matched, certain factors were difficult to match completely, such as antibiotic use and NICU duration. It is difficult to establish a normal control group in the NICU where preterm infants are admitted for treatment. Further multicenter studies and metabolome analysis might be required. Although the number of cases was limited, our study examined the changes in the intestinal microbiota of surgical NEC patients after treatment, providing some clues for further in-depth studies.

## Conclusion

In summary, our data showed that even after achieving full enteral nutrition, the alpha diversity in infants with NEC who underwent surgery was still lower than that in the control group infants. Methylobacterium, Clostridium_butyricum, and Acidobacteria were more abundant in NEC infants at the time of diagnosis and until the end of treatment. NEC infants who opted for surgery for the reestablishment and restoration of gut flora may need some more time to realize these. In addition, the pathways of the ketone body synthesis and degradation were more active in NEC patients who opted for surgery. This indicates that ketone body synthesis and degradation may be related to the pathogenesis of NEC. The pathway of sphingolipid metabolism may influence the physical development of NEC infants.

## Data Availability

The original contributions presented in the study are included in the article/**Supplementary Material**, and further inquiries can be directed to the corresponding author/s.
