# Supplementary figures and images for: Alteration of the gut microbiota after surgery in preterm infants with necrotizing enterocolitis
Source: Front Pediatr. 2023 Jan 30;11:993759. doi: 10.3389/fped.2023.993759 (PMC9923499; doi:10.3389/fped.2023.993759)

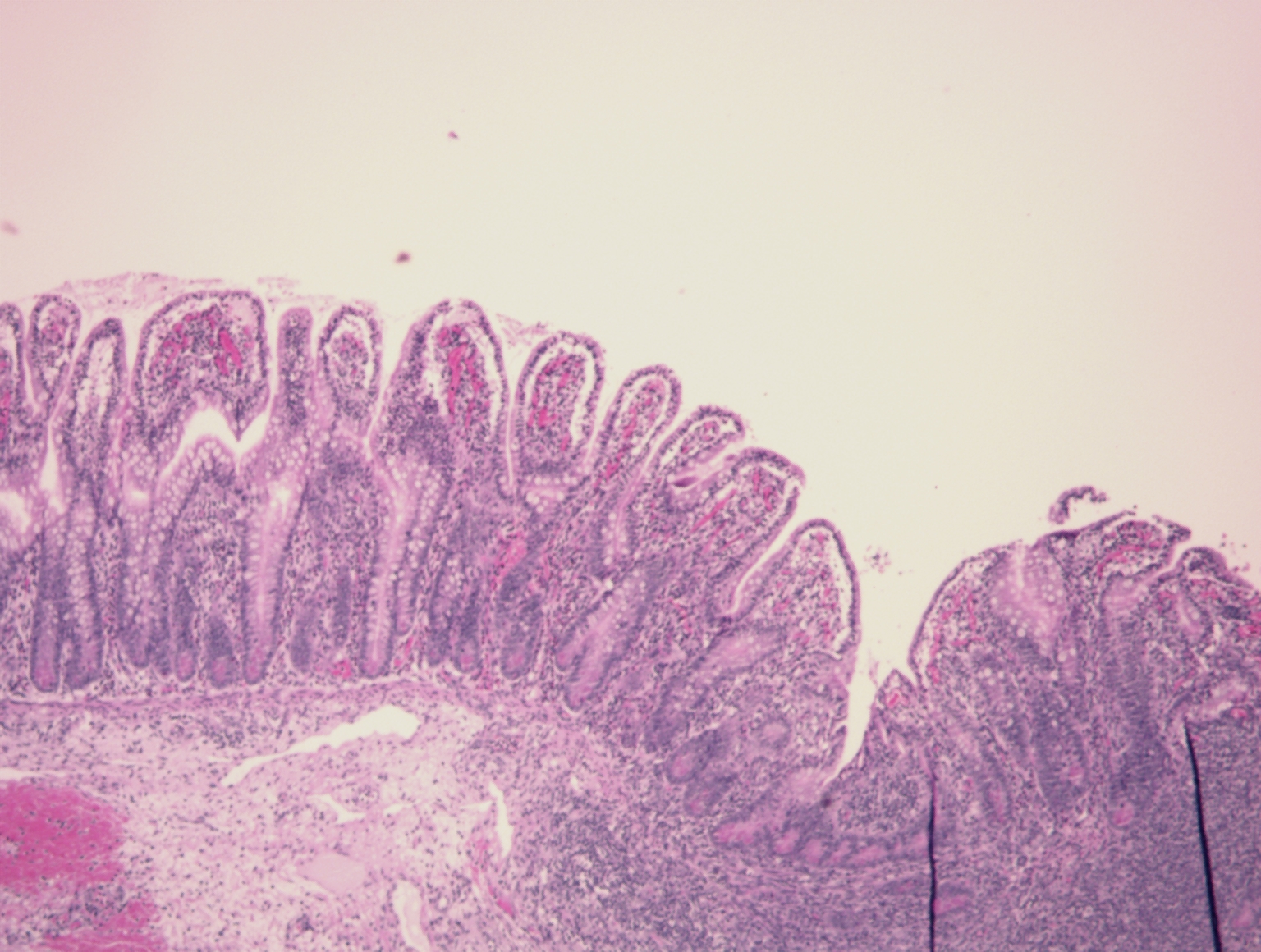

Supplement: Supplementary file 1 [file Image1.jpeg]

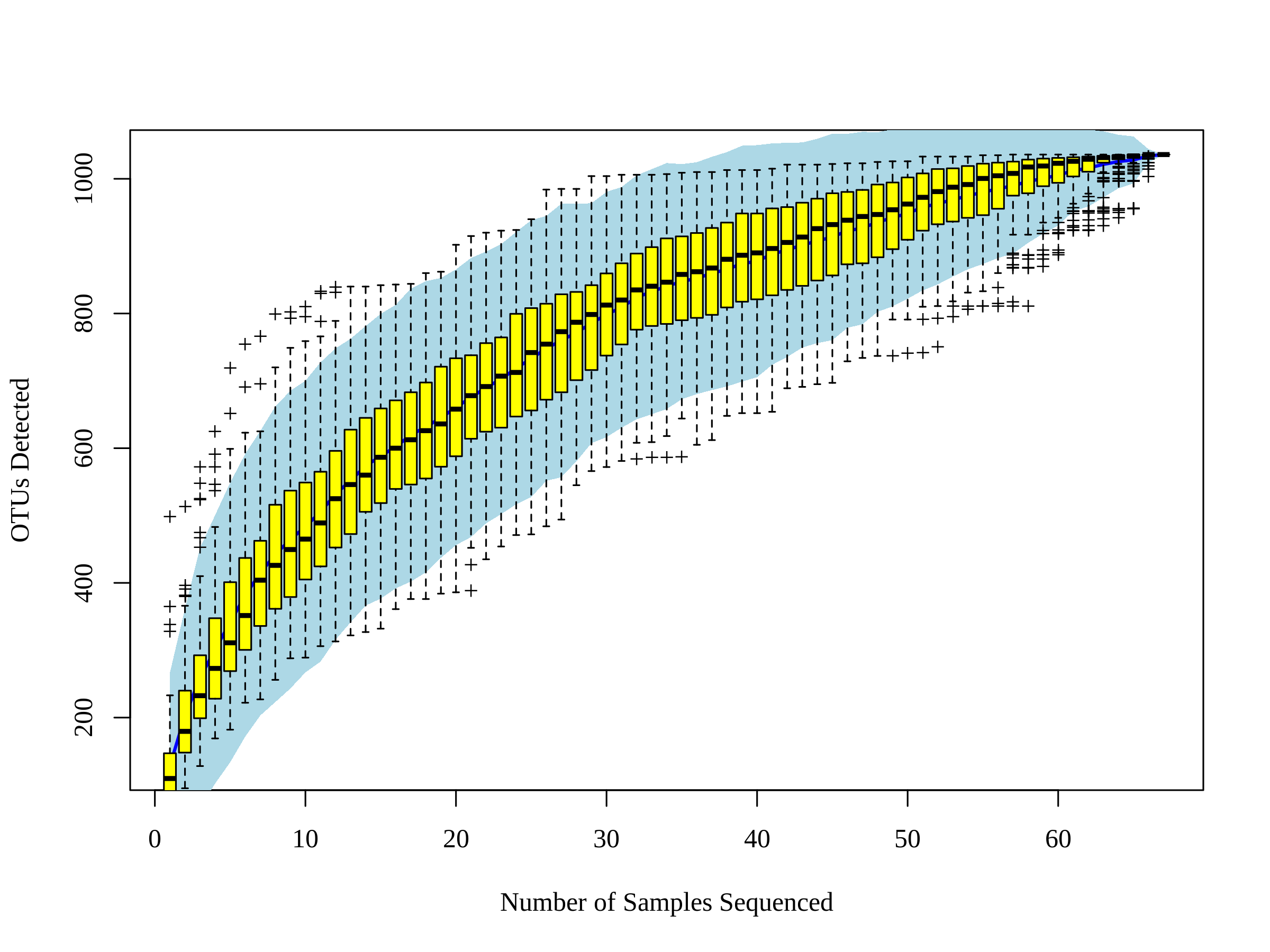

Supplement: Supplementary file 2 [file Image2.png]

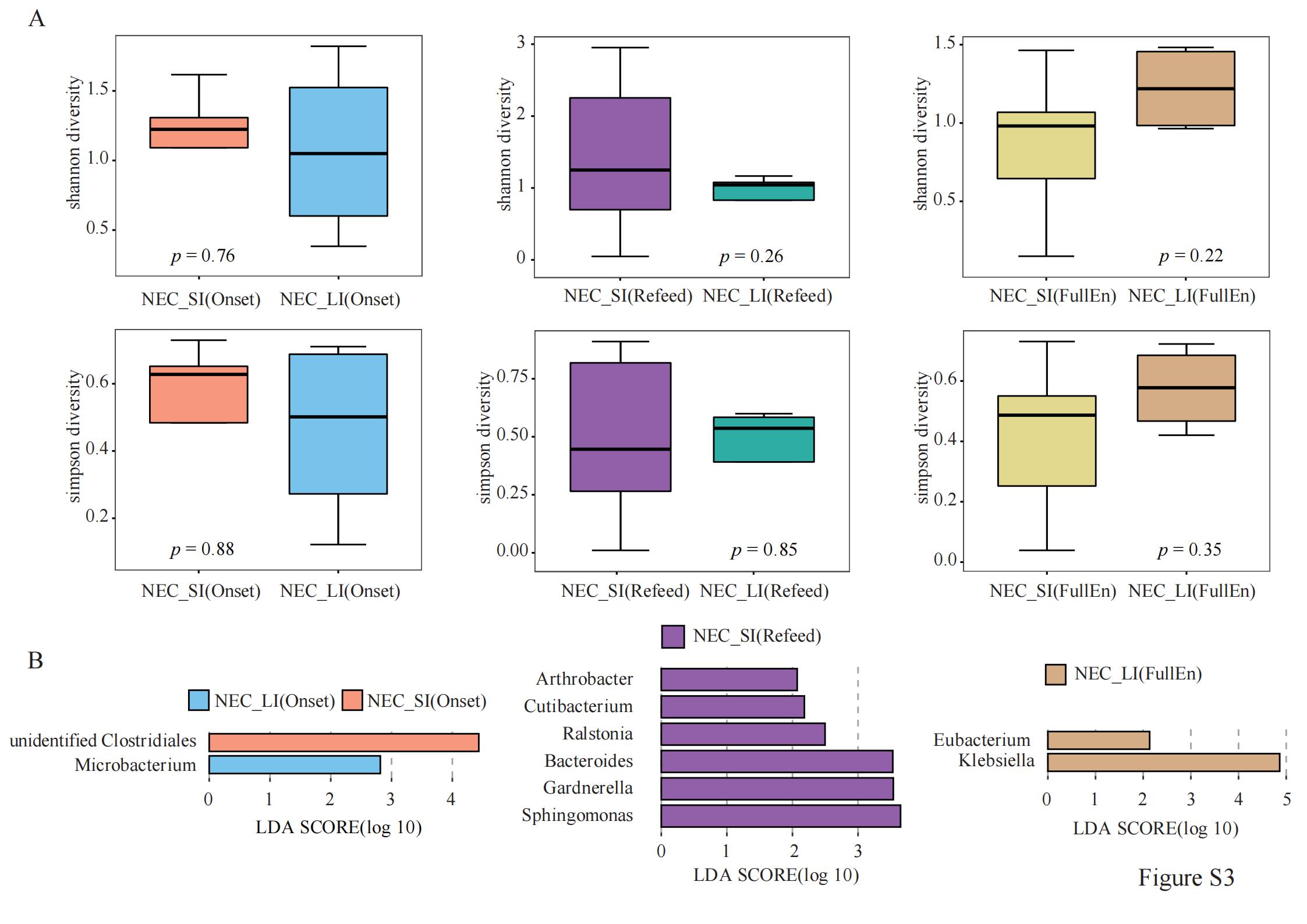

Supplement: Supplementary file 3 [file Image3.jpeg]
